# Protocol for a prospective cohort study of open tibia fractures in Malawi with a nested implementation of open fracture guidelines

**DOI:** 10.12688/wellcomeopenres.17145.1

**Published:** 2021-09-13

**Authors:** Alexander Thomas Schade, Nohakhelha Nyamulani, Leonard Ngoe Banza, Andrew John Metcalfe, Andrew Leather, Jason J. Madan, David G. Lallloo, Williams James Harrison, Peter MacPherson

**Affiliations:** 1 Public Health, Malawi-Liverpool Wellcome Trust, Blantyre, P.O.Box 30096, Malawi; 2 Liverpool School of Tropical Medicine, Liverpool, L3 5QA, UK; 3 Trauma and Orthopaedics surgery, Queen Elizabeth Central Hospital, Blantyre, BOX 95, Malawi; 4 Trauma and Orthopaedic surgery, Kamuzu Central Hospital, Lilongwe, Malawi; 5 Warwick medical school, University of Warwick, Coventry, W Midlands, UK; 6 King’s College Centre for Global Health and Health Partnerships, King's College Hospital, London, London, WC2R 2LS, UK; 7 AO Alliance, Davos, Switzerland; 8 University of Liverpool, Liverpool, L69 3BX, UK; 9 London School of Hygiene & Tropical Medicine, London, WC1E 7HT, UK

**Keywords:** injury, open fractures, low and middle income countries, implentation

## Abstract

**Background**: Road traffic injury (RTI) is the largest cause of death amongst 15–39-year-old people worldwide, and the burden of injuries such as open tibia fractures are rapidly increasing in Malawi. This study aims to investigate disability and economic outcomes of people with open tibia fractures in Malawi and improve these with locally delivered implementation of open fracture guidelines.

**Methods**: This is a prospective cohort study describing function, quality of life and economic burden of open tibia fractures in Malawi. In total, 160 participants will be recruited across six centres and will be followed-up with face-to-face interviews at six weeks, three months, six months and one year following injury. The primary outcome will be function at one year measured by the short musculoskeletal functional assessment (SMFA) score. Secondary outcomes will include quality of life measured by EuroQol EQ-5D-3L, catastrophic loss of income and implementation outcomes (acceptability, adoption, appropriateness, costs, feasibility, fidelity, penetration, and sustainability) at one year. A nested pilot pre-post implementation study of an interventional bundle for all open fractures will be developed based on other implementation studies from low- and middle-income countries (LMICs). Regression analysis will be used to model and investigate associations between SMFA score and fracture severity, infection and the pre- and post-training course period.

**Outcome:** This prospective cohort study will report patient reported outcomes from open tibia fractures in low-resource settings. Subsequent detailed evaluation of both the clinical and implementation components of the study will promote sustainability of improved open fractures management in the study sites and further scale-up of open fracture management guidelines.

**Ethics:** Ethics approval has been obtained from the Liverpool School of Tropical Medicine and College of Medicine Research and Ethics committee.

## Introduction

### Trauma in low-income countries

Road traffic injury (RTI) is the largest cause of death amongst 15–39 year old people worldwide and disability from open tibia fractures is rapidly increasing in low-income countries (LICs). Most injuries occur to the limbs and are amenable to low-cost improvement in orthopaedic care and rehabilitation ^1^. The tibia is one of the most commonly injured long bones ^2^ and due to its superficial location, it is susceptible to becoming an open tibia fracture (5.6 per 100,000 people per year in high-income countries (HICs)) ^3, 4^. Bone loss can arise from the extrusion of bone fragments at the time of injury or during surgery when devitalised segments of bone are removed, creating a defect ^5^. Open tibia fractures have devastating consequences in high-income countries but are more significant in low- and middle-income countries, including a 15% amputation rate, 18% infection rate, 15% non-union rate; only 20% of patients with open tibia fractures are able to return to work at one year ^6^. These complications have been shown to have a greater negative effect on quality of life than myocardial infarction, stroke or end-stage arthritis ^7^ and patients would be willing to give up a third of their remaining lives if they could return to good-health ^8^. Research is urgently needed to estimate the true burden of open tibia fractures, especially disability-adjusted life-years outcomes ^9^.

### Healthcare in Malawi

Malawi has a population of around 17 million people, with more than 85% living in rural areas ^10^. The gross national income per capita is about $250 (compared to an average in low-income countries of $1,313 and a global average of $11,536) ^11^. Fracture care is provided at three levels of healthcare facilities: primary care is provided in health centres; secondary care in the district hospitals (which are typically rural); and tertiary care in referral hospitals. There are four referral hospitals and one faith-based specialist orthopaedic hospital, all based in the cities of Blantyre, Zomba, Lilongwe and Mzuzu. Trained, specialised fracture care providers in Malawi include: Clinical Officers, trained to diploma and bachelor’s degree levels; and Specialist Orthopaedic Surgeons. Currently, Malawi has 12 Specialist Orthopaedic Surgeons, all based at the tertiary referral centres. There are 107 Orthopaedic Clinical Officers in clinical practice, who provide most of the fracture care in the 24 district hospitals across Malawi. Even the most severe fracture cases usually present to health centres or district hospitals first before being referred to tertiary care hospitals.

### Treatment of open fractures in Malawi

Currently, treatment of most fractures is non-operative ^12^ in Malawi and in other similar countries in sub-Saharan Africa. The reasons for use of non-operative treatment are multifactorial and include lack of trained surgeons and limited availability of implants, equipment and specialist operating theatres ^13^. Most fracture surgeries are performed in the central hospitals by orthopaedic surgeons. Even in these centres, the few available surgeons are overwhelmed with workload and opt for conservative management for some cases that could be treated operatively ^14^. As a consequence, many cases could have had better outcomes with operations ^15^.

The importance of treating open fractures was recognised by the Lancet Commission as one of the bellwether procedures that all level 1 hospitals should be able to manage ^16^. Open tibia fractures can be complicated to manage in HICs, and thus pose a real problem in a resource-limited setting ^17^. Open fractures are commonly diagnosed by clinical assessment of the wound and radiographs of the tibia, but functioning radiological services are not always available in Malawi ^18^. The most common classification is the Gustilo-Anderson ^19^ with grade III injuries having higher deep infection and amputation rates. There is, however, high inter-observer variability in its use ^20^ and other classifications have also been suggested ^21, 22^.

The management of open tibia fractures should follow established guidelines ^23^, with key components including the early administration of antibiotics, surgical ‘debridement’ (removal of all contaminated and devitalised tissue and washout of the open fracture in the operating theatre), fracture immobilisation (internal or external fixation) and application of dressings. Some studies have shown that adherence to national guidance can reduce healthcare utilisation for severe open tibia fractures in the UK ^24, 25^. Recently, open fractures guidelines have been developed in Malawi via a consensus meeting ^26^ as resources such as computed tomography and specialised orthoplastic theatre lists are often not available in Malawi.

### Outcomes of open fractures in Malawi

Follow-up of patients who have experienced fractures in Malawi is challenging due to long distances and expensive transport to health facilities ^27^. Disability or overall health measures measured by patient-reported outcome measures (PROMs) are currently unknown for open tibia fractures in LMICs ^6^. PROMs are defined as “any report of the status of a patient’s health condition that comes directly from the patient, without interpretation of the patient’s response by a clinician or anyone else” ^28^. PROs refer to patient-reported functional outcomes, such as the short musculoskeletal functional assessment (SFMA) score ^29^ or multidimensional constructs, such as health-related quality of life ^30^. To understand and improve outcomes in patient-centred healthcare systems, more patient reported outcome measures are needed to study the impact of open tibia fractures from patients’ perspectives in Malawi ^31^.

Costs to patients and the healthcare system are also key to informing policy in resource poor settings and are currently unknown for open tibia fractures in LMICs ^16, 32^. Most financial losses are not due to direct costs of medical treatment, but from indirect loss due to loss of working wages and costs of care seeking ^1, 33^. Suggested coping strategies for the financial loss included intra-family labour reallocation, use of informal loans and sale of assets ^1, 34^. Despite these coping mechanisms, a large portion of families reported secondary negative effects such as decreased food production and consumption. In Uganda, only 35% patients with long-bone injuries were able to work 12 months after injury, whereas pre-injury, 83% were working ^35^. The costs of open tibia fractures are currently unknown in Malawi and describing them will help inform public health policy.

### Training courses for open fractures in LMICs and implementation science

More than 80% of surgical, obstetric, and orthopaedic procedures could be done by associate clinicians in a practice known as “task shifting” ^16^. Task shifting in orthopaedic surgery is practised in several sub-Saharan countries ^36^. Training in key interventions such as open fracture debridement and basic fracture stabilisation (external fixator) will allow a step towards achieving the Lancet Commission’s goals of improving access to a Bellwether hospital, as more existing hospitals will be able to provide the associated procedures and will also increase the number of surgical providers and operative volume ^1^.

Some orthopaedic training courses have been adapted to LMICs, such as the primary trauma course which focuses on emergency life support ^37^. A systematic review has shown this course to improve knowledge, confidence and skills required for the effective team management of trauma and may reduce overall mortality ^38^. To our knowledge, there are no courses that have been designed to implement open fracture guidelines in a LMIC setting.

Interventions such as a training course and open fracture guidelines are not as effective if they are not implemented well. Implementation science is a form of health policy and system research that is defined as “the scientific study of methods to promote the systematic uptake of research findings and other evidence-based practices into routine practice” ^39^. It is grounded in implementation theories, frameworks and models that can classify implementation approaches into process, outcomes and evaluation ^40^. Barriers and facilitators of implementation can be categorised according to the comprehensive framework for implementation research ^41^. Implementation outcomes based on Proctor framework are an important way to conceptualise and evaluate the success or failure of implementation ^42^. There are eight defined implementation outcomes: acceptability, adoption, appropriateness, costs, feasibility, fidelity, penetration, and sustainability ^42^. These theoretical approaches have been shown to increase intervention effectiveness and reduce the “research-to-practice gap” ^43^.

### Research question

How can the outcomes of open tibia fractures be improved in a low-income country?

### Aim

To undertake a comprehensive evaluation of the management and outcomes of people with open tibia fractures in Malawi (including clinical, health system and societal factors).

### Objectives

1) To measure short-musculoskeletal function score and quality of life score over the first year from injury in a prospective cohort study among Malawian adults (aged 18 years or older) with open tibia fractures
2) To investigate the economic burden of an open tibia fracture on individuals, households, and the healthcare system in a nested economic evaluation at one year post injury
3) To pilot an open fracture quality improvement programme using implementation science strategies, measuring clinical, process and implementation outcomes

## Protocol

### Study sites

The study is a prospective observational cohort study, which will recruit all adults presenting or referred with open tibia fractures to selected hospitals over a one-year period. The data collection activity will be conducted at Queen Elizabeth (QECH) and Kamuzu Central Hospitals (KCH), as well as Dedza, Machinga, Balaka and Ntcheu District Hospitals (see Table 1). The four district hospitals were chosen as study sites because there is a high volume of trauma reported from the trauma registries in these sites ^44^. An understanding of volumes and types of fractures presenting and being treated at district level is essential to understand the national burden of open tibia fractures and current care provided.

**Table 1.** Recruitment sites’ characteristics.

Recruitment site	Characteristics
Kamuzu Central Hospital (KCH)	Central government hospital, Lilongwe (capital) 800 beds, catchment population 4 million ~60 open tibia admissions per year (2018)
Queen Elizabeth Central Hospital (QECH)	Central government hospital with NGO input, Blantyre 1000 beds, catchment population 1 million ~74 open tibia admissions per year (2018)
Ntcheu District Hospital (ND)	District government hospital, Ntcheu 255 beds, catchment population 529,000 Estimated ~15 open tibia admissions per year (2018)
Machinga District Hospital (MD)	District government hospital, Liwonde 140 beds, catchment population 300,000 Estimated ~15 open tibia admissions per year (2018)
Balaka District Hospital (BD)	District government hospital, Balaka 150 beds, catchment population 500,000 Estimated ~15 open tibia admissions per year (2018)
Dedza District Hospital (DD)	District government hospital, Dedza 150 beds, catchment population 500,000 Estimated ~15 open tibia admissions per year (2018)

### Patient population

***Inclusion criteria***

- Age ≥ 18 years
- Clinical diagnosis of open tibia fracture, made by an Orthopaedic Surgeon or Orthopaedic Clinical Officer
- Presenting or referred to either Kamuzu Central Hospital, Queen Elizabeth Central Hospital, Ntcheu District Hospital, Machinga District hospital, Balaka District Hospital or Dedza District Hospital

***Exclusion criteria***

- Patients suspected of having a fracture clinically but not confirmed by X-ray
- Patients unable/unwilling to give consent to participate

All adult patients presenting with an open tibia fracture at the selected hospitals, confirmed by X-ray, will be included in the activity. Cases will include patients with multiple fractures as well as polytrauma patients. Thus, the project will include all open tibia fractures irrespective of severity or mode of treatment (debridement, antibiotics and temporary stabilisation, definitive stabilisation and soft tissue cover).

### Primary outcome

- Change in function as measured by SMFA score between injury and one-year follow-up

### Secondary outcomes

- Change in health utility as measured by EQ-5D-3L between injury and one-year follow-up ^30^
- Incidence of infection
- Incidence of non-union ^45^

### Outcome definitions

**Infection ^46^**

- Superficial surgical site infection is defined as wound infection involving the skin and subcutaneous tissue that occurred within 30 days of surgery
- Deep surgical site infection is defined as a wound infection of the tissues deep to the skin that occurred within 90 days of injury
- Late infection is diagnosed as any late wound breakdown (>90 days) or sinus formation, or unexplained late pain with associated radiological changes consisted with peri-implant infections

**Non-union – one or both of the following:**

- Need for further surgery to achieve union ^47^
- Impaired bone healing at twelve months (RUST score < 9) ^48^

### Sample size calculation

The SMFA score has 46 items with a minimum standardised score of 0 and a maximum score of 100 per category and is comprised of two parts. Higher total scores represent greater degree of dysfunction and bother of dysfunction for certain tasks. The validation study of SMFA in Malawi reported a standard deviation of 17.3 for dysfunction index and standard deviation of 5.9 for bothersome index ^29^. Studies have reported a minimally clinically important difference of seven points for SMFA ^49^. A sample size calculation has been performed to fit a regression model that accounts for 20% relative difference in SMFA scores at one year. Assuming we have a model with 10 parameters (model terms), and we find a model that accounts for 20% of the variance in the SMFA scores, with 80% power at the 0.01 significance level (due to the multiplicity of testing), then sample size was computed using R version 4.0.3 (10 variables, effect size = 0.2/(1-0.2), significance level = 0.01, power = 0.8). Calculation showed 100 participants will be required for the definitive analysis. Allowing for 20% loss to follow-up, for the cohort study, the sample size required is 125 participants to detect a 20% relative difference. Potential confounders include gender, site of recruitment, presence of co- morbidities and other injuries.

### Economics study

This will estimate economic burden based on a patient, health system and societal perspective. For the participant costs, all patients in the prospective cohort study across all six study sites and at each follow-up will complete a patient costing questionnaire adapted from the World Health Organization (WHO) tool for assessing tuberculosis catastrophic costs and palliative patient costing care done in Malawi ^50, 51^.

Hospital costs will be collected using a micro-costing methodology previously used for femoral fractures in Malawi ^52^. Resource identification will be done by a focused time & motion analysis of participants. Staff and resources that are involved in the care of patients will be identified. For the staff salaries, leave days, public holidays and average sick days for this cadre will be removed.

Direct costs will be quantified for procedures and ward personnel, medications, laboratory and radiology investigations, surgical implants, procedure instruments and disposable supplies. Supplies will include intraoperative medications, disposables, intravenous fluids, blood products, and others. The mean cost of supplies for intramedullary nailing, skin grafts and skin flaps will be calculated by multiplying prices by utilization. The average number of patients per day will be calculated by dividing the average monthly inpatient days by 30. Type and quantity of medications and investigations will be recorded from the patient chart. The labour and resource costs of radiographs and laboratory investigations will be obtained from the radiology department and the hospital laboratory. Average cost per patient will then be calculated for each intervention.

Indirect overhead costs will include food, building maintenance, renovation, cleaning and sanitation, beddings, stationery, uniforms and protective gear, staff training and maintenance. These will be obtained from the hospital accountant and procurement office.

### Primary outcome

- Direct and indirect costs from a societal perspective of open tibia fractures in Malawi

### Secondary outcomes

- Economic impact to the patient and their household
- Economic coping mechanisms of the patient and their household
- Length of stay in hospital
- Costs per patient from a health system perspective

### Sample size calculation

This is descriptive and no sample size calculation was performed.

### Implementation study

There will also be a pilot pre-post implementation study of an interventional bundle for all open fractures based on other implementation studies from LMICs ^45, 46^. The intervention bundle will occur six months after the start of recruitment (see Table 2). The training course will be developed locally with stakeholder input and participatory action research. We shall interview healthcare professionals (doctors, nurses, and clinical officers) before the course, immediately after the course and one year after the course to assess implementation outcomes.

**Table 2.** An outline of the core and adaptable components of the interventional bundle.

Core components
• A hospital management protocol for all open fractures will be implemented at first and second level healthcare facilities based on the 17 Malawi Orthopaedic Association/AO (Arbeitsgemeinschaft für Osteosynthesefragen) Alliance guidelines ^26^. This protocol will include administering early antibiotics, photographing the wound and performing debridement in theatre with adequate anaesthesia • A national educational course for clinical officers on the open fractures guidelines • Improved documentation of each guideline by using standard proformas for open fracture management • Transferring grade III open fractures to central hospitals as soon as possible and keeping wound open for grade IIIB and IIIC injuries
Adaptable components
• Dissemination of guidelines via posters in all the primary and secondary centres that may refer to tertiary centres +/- back-up to referral centres • In addition to the above, site visits to selected referral centres for education and training to enhance implementation of the protocol. Visits and training will be undertaken by local MDT members and the principal investigator. • Sharing of healthcare telephone contacts to improve communication of referrals to tertiary centres • A letter signed by hospital director and the chair of the Malawi Orthopaedic Association encouraging that open fractures should be debrided in theatre with spinal or general anaesthesia • Feedback for the healthcare professionals in the first 3 months after the course on pre-post debridement photos • Radio and/or television campaign

### The training course

The open fracture training will be determined by nominal group technique to gain consensus on: 1) the topics to include, 2) the pre-course material 3) modes of delivery and 4) the evaluation methods. The group will include experts in surgical system strengthening (or health systems research), clinical orthopaedics and educationalists to comment on pedagogical aspects. Advantages and disadvantages of available options will be discussed and then each members will vote anonymously to choose the best option/s. The final course programme will be agreed with national stakeholders in the Malawi Orthopaedic Association.

### Primary outcome

- Change in function as measured by the Short Musculoskeletal Function Assessment (SMFA) between injury and one-year follow-up

### Secondary outcomes

- Compliance with the Malawi Orthopaedic Association/ Arbeitsgemeinschaft fur Osteosynthesefragen Alliance open fracture guidelines ^26^
- Rate of complications including infection and non-union
- Implementation outcomes including the acceptability (AIM) score, appropriateness (IAM) score, feasibility (FIM) score and NoMAD validated survey

### Stakeholders

Malawi Orthopaedic Association/AO Alliance/Ministry of Health. The training course will be aimed at all 107 orthopaedic clinical officers that attend the Malawi Orthopaedic Association annual conference.

### Sample size

A multivariate linear regression model will be used to compare average SMFA indices (dysfunction and bothersome) ^53^ at one year post injury for patients recruited pre- and post-intervention. We find a model that accounts for 20% of the variance in the SMFA scores, with 80% power at the 0.05 significance level. Sample size was computed using R. Calculation showed 128 participants will be required for the definitive analysis. Allowing for 20% loss to follow-up, for the cohort study, the sample size required is 160 participants to detect a 20% relative difference in SMFA scores at 1 year. A sample size of n=80 per group (80 participants before training course and 80 participants after) is required to detect a minimum clinically important difference with 80% power, with 20% drop-out.

### Validity and reliability

The project coordinator and the investigators will supervise the data collection and regularly liaise with the clinicians at each site to identify and rectify any problems. Such regular supervision will also ensure maintenance of high standards of data collection. At random visits on each site, the project coordinator will tally the collected forms to cases in the patients’ register book and record the number of missed cases. In addition, a senior investigator will assess completion of all collected forms and any problems identified will be discussed with the responsible personnel either through telephone or during the supervisory visits.

### Data analysis

Regression analysis will be used to model and investigate associations between fracture severity and key study outcomes (adjusted for confounders including age and sex and for baseline values). Regression analysis will also be undertaken to evaluate the implementation outcomes including the acceptability (AIM) score, appropriateness (IAM) score, feasibility (FIM) score and fidelity on key variates especially Gustilo grading (fracture severity). Analysis of change of scores before and after (including baseline values in model as a covariate) will also be used to assess EQ-5D and SMFA during the post-implementation phase to determine if there is an upwards, stable or downwards trend following implementation. We will use the Multiple Imputation Chained Equations (MICE) and the AMELIA programme in R statistical software version 4.0.3. Regression coefficients from multivariable regression will be combined across multiple multiply imputed datasets using Rubin’s rules.

### Data management

Anonymous, de-identified patient data will be entered into REDCap version 11.0.3 by the study teams. At no stage will the principal investigator or any team members outside of the local study centre have access to the key to the pseudo-anonymised data. The Malawi-Liverpool-Wellcome Trust have standard operating procedures that will be followed in relation to data handling, management, storage, consent, and ethics. Data will be created and stored in accordance with all applicable UK, European and Malawian data protection regulations and best practice guidelines – including ICH Good Clinical Practice and Clinical Data Acquisition Standards Harmonisation (CDASH) guidelines. Study participants will be assigned a unique study number at the time of recruitment, under which all electronic data will be captured. From this point, all data will be stored in anonymised form. Data will be collected and stored, using online-based data-capture technologies.

### Data collection

Data will be collected via electronic case report forms using REDCap electronic data capture tool. The data will be entered by research staff directly into REDCap using small tablets. Data collected at enrolment include details of the injury, pre-existing medical conditions, work status and method of treatment. Intraoperative data, such as the type and size of implants used are to be entered by staff with assistance from one of the investigators using the operative note in the medical record. In addition, each subject will verbally complete the Chichewa translated EurooQoL EQ-5D and SMFA questionnaires. To estimate the preoperative quality of life and musculoskeletal function, subjects will be asked to recall function prior to injury. This has been shown to be a valid method for establishing a baseline health-related quality of life post-operatively in arthroplasty populations ^54^.

The electronic data will be stored on the MLW servers, which will be password protected and only the research team will have access to the password. A fully anonymised dataset will be encrypted, and password protected on laptops for analysis only.

The only data not initially entered into REDCap are the injury and post-operative radiographs, which are photographed with a digital camera using a mobile phone (Samsung, Suwon, South Korea). Any patient-identifying information will be hidden before photographs are taken. This allows seamless upload to a web-based repository using a pre-built mobile-based form application (Open Data Kit). Baseline radiographs are reviewed to determine Gustilo, Orthopaedic Trauma Association & Orthopaedic Trauma Society fracture classification ^54^, post-surgery angulation at the fracture site and bone loss. These data will also be recorded using REDCap version 11.0.3.

All study materials including consent form and assessment tools can be found as *Extended data* ^55^.

### Ethical considerations

Ethical approval has been gained at all participating sites. Ethical approval reference numbers: Liverpool School of Tropical Medicine Research Protocol (20-068) and College of Medicine Research and Ethics Committee P.09/20/3130.

### Ethical issues

The patient reported data will collect anonymous data. All data will be handled in strictest confidence and patients will not be identified in any of the reports produced from the activity. Only the project team will have access to the keys for the filing cabinets, a password for the computer and the external drive containing the data and will not disclose the password or share any part of the data with anyone. All research staff will undergo Good Clinical Practice (GCP) training. Compliance with regulations and legislation in Malawi as well as research best practice will be monitored in conjunction with the MLW Clinical Research Support Team.

The research assistants will regularly attend the surgical wards to identify participants that would meet the inclusion criteria. They will approach the patient in the first 24–48hrs of admission as some less severe injuries may be discharged within this timeframe. A patient information leaflet and consent form will be given to all participants ^55^. Should they have any queries or concerns, the research team will be available to answer them. Informed participant consent (either written or independently witnessed thumbprint if illiterate) will be collected by a trained research assistant. Paper consent forms will be stored securely, initially on site at the recruitment site and then transported weekly to MLW for secure storage. There are no children in this study and, therefore, parental consent is not required. If the patient has an irreversible head injury, they will be excluded from the study. If they are temporarily unable to give consent, then they will be recruited once they have regained the capacity to consent.

### Dissemination

Results of this study will be used to provide a baseline in order to investigate different treatment options for open tibia fractures appropriate for Malawi and similar settings. The economic data produced will act as a key metric for injury that can be incorporated into future trials. Particularly in a limited resource environment, these skills will be key to assess the cost-effectiveness of interventions and, ultimately, to improve policy adoption.

The findings of this study will also be compiled into a written report which will be made available to each participating hospital and College of Medicine Research Ethics Committee (COMREC). In addition, a copy of the report will be made available to the Ministry of Health headquarters as the findings may have an impact in future planning and policies in terms of resource allocation and formulation of intervention strategies. An abstract will be presented to national and international conferences and one or more articles will be submitted to open-access peer-reviewed journals for publication.

### Study status

This study is currently recruiting.

## Discussion

Disability measured by patient reported outcomes should be used to evaluate health care interventions and complement conventional outcome measures such as mortality, as they may demonstrate the impact of injury on other areas of life from the patient’s perspective. Currently, there is little research on assessing function, quality of life and economic outcome of patients with diaphyseal open tibia fractures. This is needed to understand important factors for treating these injuries.

There are some limitations to the study. This study might not represent all the open tibia fractures in Malawi. We acknowledge this might not represent the situation in northern Malawi, however, as there are no orthopaedic surgeons in northern Malawi, some patients may be referred to Kamuzu Central Hospital. In addition, the recruitment sites are located in the central and southern regions of Malawi where there is more concentrated population with a major motorway connecting both. The SMFA score includes questions about working, driving, self-care, sexual activity and the Chichewa version was only validated in the adult population ^29^. Children and adolescents are therefore excluded in our study; however, open fractures are much more common in the adult population.

Heterogeneity of open tibia fractures may reduce the power of our analysis. Some advocate for Gustilo type I injuries to be treated as closed injuries, however, there is growing evidence that Grade I injuries have higher rates of infection than reported and should be treated aggressively. We will conduct a sub-analysis, if possible.

The intervention will be determined by an in-country nominal group technique but will include key components of the MOA/AO Alliance open fracture standards and guidelines which were determined before the study ^26^. There are no other open fracture management guidelines in other LMICs, but it is unknown if these are generalisable to other LMICs. Our intervention might bias the outcomes of our cohort study, but this pilot intervention will provide evidence for larger interventions in the future. A balance was reached between describing the injury and providing a small pilot intervention. The Hawthorne effect might also bias the primary outcomes, but the implementation outcomes based on healthcare professional questionnaires might help understand whether guidelines have been effectively implemented.

This study will develop an effective academic-practice-policy maker partnership to translate evidence from open fracture management into practice and national policy. Government officials regularly attend conferences where we present our data and they are keen to engage with us to translate research findings into policy. Standardisation of open fracture care in HICs has resulted in improved patient outcomes and we hope this can be replicated in Malawi and other LICs, as referrals between district and central hospitals are currently not well streamlined. These strengthened collaborations will also provide a platform for future research.

## Data availability

### Underlying data

No underlying data are associated with this article.

### Extended data

Open Science Framework. TITAN protocol. https://doi.org/10.17605/OSF.IO/N36EG ^55^.

This project contains the following extended data:

- Data capture forms.docx
- Implementation tools.docx
- TITAN Chichewa PIL.docx (Patient information leaflet)
- TITAN English PIL.docx (Patient information leaflet)
- TITAN protocol manuscript 5.docx

Data are available under the terms of the Creative Commons Zero "No rights reserved" data waiver (CC0 1.0 Public domain dedication).
