# Supplementary material for: Protocol for a prospective cohort study of open tibia fractures in Malawi with a nested implementation of open fracture guidelines
Source: Wellcome Open Res. 2021 Sep 13;6:228. [Version 1] doi: 10.12688/wellcomeopenres.17145.1 (PMC9034175; doi:10.12688/wellcomeopenres.17145.1)
Supplement: Peer review history

# Peer review: Reviewer response for version 1

**DOI:** 10.21956/wellcomeopenres.18938.r49501

**Published:** 2022-04-22

**Authors:** Dominic Konadu-Yeboah

**Affiliations:** 1 Directorate of Trauma and Orthopaedics, Komfo Anokye Teaching Hospital, Kumasi, Ghana; 2 Department of Surgery, School of Medicine and Dentistry, Kwame Nkrumah University of Science and Technology, Kumasi, Ghana

I have reviewed the paper entitled ‘Protocol for a Prospective Cohort Study of Open Tibia Fractures in Malawi with a nested Implementation of Open Fracture Guidelines’. The paper uses a prospective cohort design to investigate the disability and economic outcomes of patients with open tibial fractures in a low-income country and how to improve these indices with the implementation of open fracture guidelines. The study involves participants drawn from six trauma care centres in an African country and measures outcomes of open tibial fractures. The study question is appropriate and has the potential of contributing immensely to knowledge in the management of open fractures of the tibia in resource-limited settings.

 There are a few minor suggested corrections:

1. Under 'Treatment of open fractures in Malawi', the statement "Open fractures can be complicated to manage is unclear", could be reframed as, for example, "The management of open fractures can be challenging".
2. Outcomes of open fractures in Malawi:
  - The statement "expensive transport to health facilities" is clumsy and could be reframed for example as "it is expensive to travel to health facilities".
  - "Disability or overall health measures measured by patient-reported outcomes measures...", the word 'measure' has appeared three times in succession making the statement clumsy, could it be reframed?
3. Table 1: "Open tibia admissions per year" should be reframed as "open tibia fracture admissions per year".
4. 'The training course': "Advantages... and then each members will vote", should read, "advantages... and each member will vote".

Overall, the protocol is scientifically sound.

Is the study design appropriate for the research question?

Yes

Is the rationale for, and objectives of, the study clearly described?

Yes

Are sufficient details of the methods provided to allow replication by others?

Yes

Are the datasets clearly presented in a useable and accessible format?

Yes

Reviewer Expertise:

Orthopaedics and Trauma

I confirm that I have read this submission and believe that I have an appropriate level of expertise to confirm that it is of an acceptable scientific standard.

# Peer review: Reviewer response for version 1

**DOI:** 10.21956/wellcomeopenres.18938.r46408

**Published:** 2021-10-18

**Authors:** Chris Lavy

**Affiliations:** 1 Nuffield Department of Orthopaedics, Rheumatology and Musculoskeletal Sciences, Botnar Research Centre, University of Oxford, Oxford, UK

This is a prospective study looking at the outcomes of 160 patients with open tibial fractures treated according to existing practices. The study will have two components, the first component looking at the outcome using patient reported outcome and functional scoring. The second using economic tools assessing the cost to the hospital/state, and the economic effect/burden on the individual and his/her family. 

 The results of the study will be used to design and then pilot a quality improvement programme using implementation science strategies. 

**Strengths:**

- The study looks at the situation as it is in Malawi, with the manpower and infrastructure that actually exists. Many studies have compared conservative to operative treatment and come up with the conclusion that surgical is better. But such a conclusion is almost impossible to implement in a country where most hospitals don’t have a surgeon, let alone an orthopaedic surgeon or facilities for internal fixation.
- The Protocol is clear and in my opinion manageable in all the trial sites. Clear definition of the clinical variables has been decided. Eg definitions of infection and non union.
- The sample size and power calculations show that it is likely that the study will lead to significant findings. 
- The economic study is novel and will be of benefit to government, NGOs, WHO and health planners in Malawi and the region.
- The quality improvement programme will have a robust evidence base, and will also have ownership by clinicians.

**Weaknesses:**

- A possibility that the cohort might not be representative of all tibial open fractures.
- Data capture and storage. This is always a risk in rural hospitals which are not used to running trials.
- The Hawthorne effect as clinicians’ day to day decisions and practice will be recorded and discussed.

**Opinion:**

 The authors have chosen an area of enormous need where there has been little well organized research. The unnecessary death, disability and financial loss due to road traffic accidents in LMICs particularly in Africa is under researched, and under funded. The potential for constructive output from a project like this in terms of policymaking and education is huge. The project is well thought out. The investigators are all experienced in LMIC work and have taken a focused and practical approach which also has scientific rigour. They are aware of the weaknesses that I have outlined and have taken an honest approach to mitigating them as far as possible. I would support this study and have no specific changes to recommend.

Is the study design appropriate for the research question?

Yes

Is the rationale for, and objectives of, the study clearly described?

Yes

Are sufficient details of the methods provided to allow replication by others?

Yes

Are the datasets clearly presented in a useable and accessible format?

Yes

Reviewer Expertise:

Trauma and orthopaedic surgery

I confirm that I have read this submission and believe that I have an appropriate level of expertise to confirm that it is of an acceptable scientific standard.
